# *ADNP*-Related Neurodevelopmental Disorder: The First Turkish Case Series with Novel Variants and Reduced Intrafamilial Penetrance

**DOI:** 10.3390/diagnostics16172879

**Published:** 2026-09-07

**Authors:** Ahmet Kablan, Abdullah Sezer, Atakan Deniz, Elifcan Taşdelen, Firdevs Dinçsoy Bir, Abdulkerim Kolkıran, Melike Ataseven Kulalı, Kerem Teralı, Emregül Işık

**Affiliations:** 1 Department of Medical Genetics, Ankara Etlik City Hospital, Ankara 06170, Türkiye; abdullahsezer25@gmail.com (A.S.); atakandenizmd@gmail.com (A.D.); elifkarakaya2012@gmail.com (E.T.); firdevsdincsoy@gmail.com (F.D.B.); 2 Department of Pediatric Genetics, Ankara Etlik City Hospital, Ankara 06170, Türkiye; kolkiranaa@gmail.com (A.K.); melikeataseven-81@hotmail.com (M.A.K.); 3 Department of Medical Biochemistry, Faculty of Medicine, Cyprus Health and Social Sciences University, Morphou 99750, Cyprus; keremterali@gmail.com; 4 Department of Pediatric Endocrinology, Bilkent City Hospital, Ankara 06800, Türkiye; dremregul@gmail.com

**Keywords:** Helsmoortel–Van der Aa syndrome, novel variants, reduced penetrance Turkish case series

## Abstract

**Background/Objectives:** Helsmoortel–Van der Aa syndrome (HVDAS), or ADNP syndrome, is a neurodevelopmental disorder characterized by cognitive deficits, dysmorphic features, and multisystem involvement. While typically arising de novo, familial cases with variable penetrance remain exceptionally rare. No cases have been reported from Türkiye to date. This study aims to define the first Turkish case series and provide compelling evidence for familial inheritance with variable penetrance/expressivity. **Methods:** We clinically and molecularly evaluated six Turkish subjects from four unrelated families presenting varying degrees of clinical display such as developmental delay, intellectual disability, or autism spectrum disorder. Next-generation clinical exome sequencing was performed, followed by Sanger sequencing for variant validation and parental segregation analysis. **Results:** Four distinct heterozygous truncating/frameshift variants were identified (three of them were novel) across the cohort, all falling within the CpG hypomethylating epigenetic group. Notably, a familial cluster (three individuals) shared the c.2815_2816del p.(Ile939Serfs*4) variant. Within this family, striking intrafamilial variability and reduced penetrance were observed: the 2.5-year-old proband displayed a severe phenotype with epilepsy and brain abnormalities, his 17-year-old sister showed a remarkably mild phenotype, and their 47-year-old father was almost entirely asymptomatic. Additionally, prolonged umbilical cord retention complicated by infection was identified as a novel clinical finding in two unrelated probands. **Conclusions:** This study presents the first Turkish case series of ADNP syndrome, expanding its global mutation spectrum with novel variants. Our findings provide robust evidence for familial transmission with reduced penetrance, emphasizing the necessity of family-based genetic counseling and parental testing, even when evaluating apparently asymptomatic individuals.

## 1. Introduction

Helsmoortel–Van der Aa syndrome (HVDAS), also known as *ADNP* syndrome (MIM #615873/MIM *611386), is an abnormality characterized by a neurodevelopmental (cognitive and behavioral) spectrum, craniofacial/dysmorphic features, and extra-neurological manifestations involving the gastrointestinal, cardiac, immune, and skeletal systems. Since its first description in 2014, *ADNP* has emerged as one of the most frequently mutated single genes in autism spectrum disorder and developmental delay cohorts [1].

*ADNP* encodes a transcription factor and chromatin regulator that plays a critical role in neuronal development, synaptic formation, and neuroprotection [2,3]. Most reported pathogenic variants are de novo truncating mutations, particularly clustered in the C-terminal region of the protein, supporting a haploinsufficiency-related disease mechanism, even though missense alterations have also been reported recently [4]. However, increasing use of next-generation sequencing has revealed greater molecular and clinical heterogeneity than initially appreciated [5]. Currently, the largest group published in the literature belongs to the study by Van Dijck et al., which described 78 patients. In addition, a more recent systematic review evaluated a total of 105 cases reported across 34 publications [5,6].

Although *ADNP*-related disorder has been reported in diverse populations, no cases from Türkiye have been described to date, leaving a significant geographic and ethnic gap in the literature. Moreover, while the disorder is generally considered to follow a de novo dominant inheritance pattern, very rare familial transmissions and instances of variable expressivity and reduced penetrance have been reported, especially in the C-terminus, raising important questions about disease mechanisms and genetic counseling [5].

In this study, we report six genetically confirmed Turkish patients with *ADNP*-associated neurodevelopmental disorder. Notably, three of the identified variants are novel, and none of the mutations correspond to previously reported recurrent hotspots. In addition, we describe an unusual family in which two affected siblings (an adolescent girl and a 2-year-old boy) inherited the same *ADNP* variant from their father, who is clinically unaffected aside from obesity, providing compelling evidence of reduced penetrance and intrafamilial variability.

## 2. Materials and Methods

This study includes six individuals diagnosed with *ADNP*-associated neurodevelopmental disorders. Patients were referred for genetic evaluation due to developmental delay, intellectual disability, autism spectrum disorder, and/or syndromic features. Two of the patients were siblings from the same family, and their father was also evaluated following the detection of a shared *ADNP* variant.

All participants or their legal guardians provided written informed consent for genetic testing and the use of anonymized clinical and molecular data for research purposes. The study was conducted in accordance with the Declaration of Helsinki and approved by the local institutional ethics committee. This study protocol was reviewed and approved by the Local Ethical Committee of Ankara Etlik City Hospital in Ankara, Türkiye (approval number: AEŞH-BADEK1-2026-109).

Genomic DNA was extracted from peripheral blood leukocytes using standard methods following the manufacturer’s instructions. All probands underwent clinical exome sequencing (CES) using a targeted exome capture kit covering the coding regions and exon–intron boundaries (Clinical Exome Solution By Sophia Genetics kit, mitochondrial DNA sequencing included) on the NovaSeq Platform (Illumina, San Diego, CA, USA) for next-generation sequencing analysis (NGS). Sequencing quality was assessed based on overall mean depth, the percentage of target bases covered at ≥20×, and coverage of the *ADNP* gene. The mean sequencing depth was 108×, with 97.8% of target regions covered at ≥20×, while the *ADNP* coding region and exon–intron boundaries achieved [99.1%] coverage at ≥20×. Potential causative variants that had <20× reads on NGS were confirmed with Sanger sequencing before reporting. Sanger sequencing was also performed for family segregation and confirmation of co-segregation in parents (ABI 3500xl Genetic Analyzer Applied Biosystems, Foster City, CA, USA).

Variant prioritization focused on rare variants (minor allele frequency <1% in population databases) located in genes associated with neurodevelopmental disorders. Variants in *ADNP* were evaluated in detail based on predicted functional impact, inheritance pattern, and consistency with the patients’ clinical phenotypes (NM_001282531). Variant pathogenicity was classified according to the American College of Medical Genetics and Genomics (ACMG) [7]. Novel variants were defined as those not reported in ClinVar, HGMD, or gnomAD at the time of analysis. Variants located within the coding region from c.2000 (p.667) to c.2340 (p.780) were classified as class II (epi-ADNP-2 profile, characterized by CpG hypermethylation), whereas variants outside this interval were classified as class I (epi-ADNP-1 profile, characterized by CpG hypomethylation). 3D structural model of the human ADNP protein generated by AlphaFold (model identifier: AF-Q9H2P0-F1-model_v6). 2D linear schematic illustrating the domain organization of the same ADNP protein from the N-terminus to the C-terminus, prepared using IBS 2.0 (Illustrator for Biological Sequences).

## 3. Results

### 3.1. Patient #1

**Figure 1:**
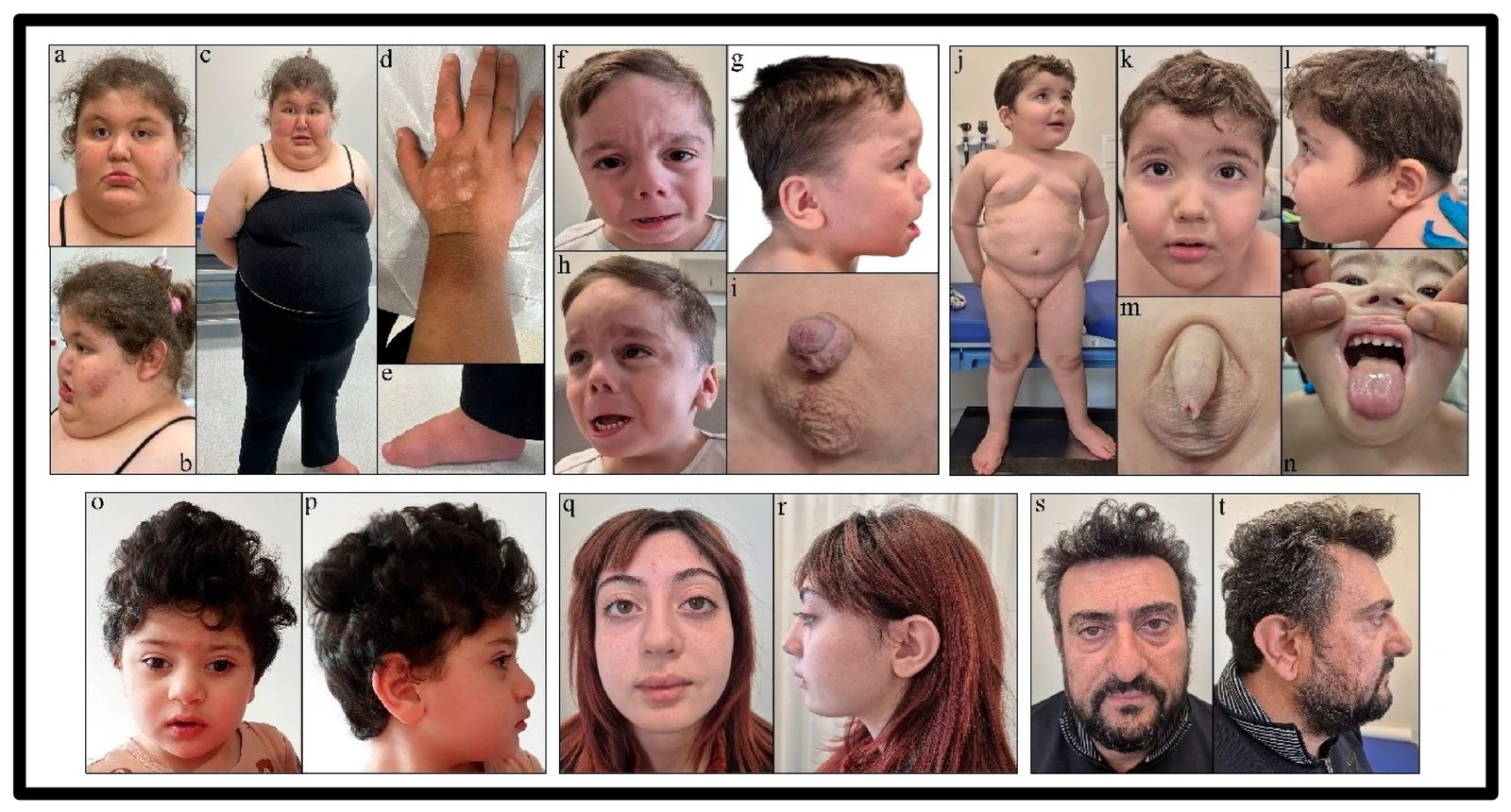
Dysmorphic and associated clinical features of the six patients. (**a**,**b**) Frontal and lateral facial views of Patient 1. (**c**) Generalized obesity. (**d**) Scar formation over the dorsum of the hand secondary to chronic self-injurious biting behavior. (**e**) Pes planus. (**f**,**g**) Frontal and lateral facial views of Patient 2. (**h**) Multiple diastema. (**i**) Genital hypoplasia. (**j**) Obesity phenotype of Patient 3 (**k**,**l**) Frontal and lateral facial views of Patient 3. (**m**) Genital hypoplasia. (**n**) Multiple diastema. (**o**–**t**) Frontal and lateral facial views of the familial subjects respectively (Patients 4–5–6). (**o**,**p**) strabismus, upslanting palpebral fissures, broad nasal bridge, low anterior hairline, short columella, synophrisis. (**q**,**r**) and (**s**,**t**) show minimal or no facial dysmorphism.

**Figure 2:**
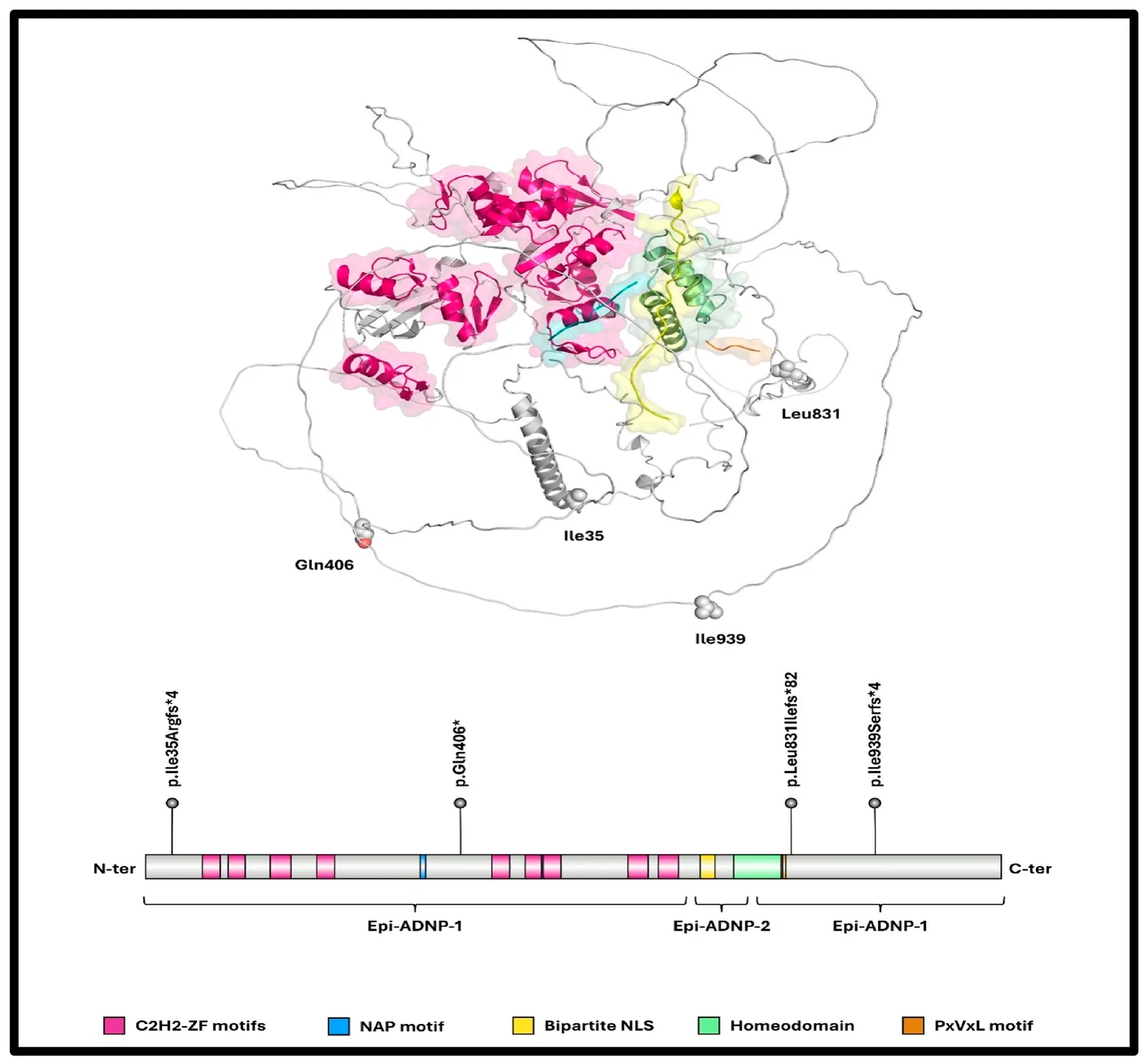
Structural and linear domain representation of the human ADNP protein. **Top panel:** A 3D structural model of the human ADNP protein generated by AlphaFold (model identifier: AF-Q9H2P0-F1-model_v6) [8]. The structure is represented as a ribbon diagram with a semi-transparent surface overlaid on the functional domains. Gray spheres mapped onto the 3D structure indicate the specific amino acid residues (Ile35, Gln406, Leu831, and Ile939) where identified genetic variants occur. **Bottom panel:** A 2D linear schematic illustrating the domain organization of the same ADNP protein from the N-terminus to the C-terminus, prepared using IBS 2.0 (Illustrator for Biological Sequences) [9]. The schematic shares the same color-coding scheme as the 3D model above to map specific functional regions (magenta: Cys2His2-type zinc-finger [C2H2-ZF] motifs; blue: NAP motif; yellow: bipartite nuclear localization signal (NLS); green: homeodomain; orange: PxVxL motif). Lollipop markers on the linear sequence denote the positions of four specific truncating/frameshift variants (p.Ile35Argfs*4, p.Gln406*, p.Leu831Ilefs*82, and p.Ile939Serfs*4). Below the sequence, brackets indicate regions corresponding to distinct DNA methylation episignatures associated with ADNP syndrome. The N-terminal and C-terminal regions map to the epi-ADNP-1 profile (CpG hypomethylation), while the central region containing the NLS maps to the epi-ADNP-2 profile (CpG hypermethylation).

A 13.5-year-old female was diagnosed at 11 years of age. She presented with severe obesity and macrocephaly. Neurodevelopmental evaluation revealed significant motor delay and severe speech delay, with no sentence formation and limited vocabulary. Dysmorphic features included low anterior hairline, hypertelorism, depressed nasal bridge, short nose, sparse eyebrows, epicanthal folds, narrow palpebral fissures, everted ear lobules, long philtrum, and thin upper lip. Skeletal findings included brachydactyly, pes planus, and short neck (Table 1, Figure 1a–e). She had a history of secundum atrial septal defect requiring surgical closure. Additional findings included hepatomegaly, hepatosteatosis, metabolic syndrome, and behavioral abnormalities such as hand stereotypies and self-injurious behavior. Brain imaging demonstrated nonspecific diffusion abnormalities. Notably, she had prolonged umbilical cord retention complicated by infection. Genetic analysis identified a heterozygous frameshift variant in the *ADNP* (c.2491_2494del; p.Leu831Ilefs*82) (Table 2, Figure 2).

### 3.2. Patient #2

A 4-year-old male, diagnosed at 2 years of age, presented with motor delay and severe speech delay with absence of meaningful words. Early neuroimaging showed mild ventriculomegaly that normalized over time, while later imaging revealed delayed myelination and posterior white matter abnormalities. Dysmorphic features were also noted alongside genital anomalies (micropenis and scrotal hypoplasia) (Table 1, Figure 1f–i). Cardiac evaluation revealed a bicuspid aortic valve and patent foramen ovale. He had recurrent infections, bilateral inguinal hernia, and cryptorchidism requiring orchiopexy. Behavioral features included absent speech and a family history of speech delay. He also exhibited early tooth eruption and prolonged umbilical cord retention with omphalitis requiring hospitalization, complicated by cardiac arrest in infancy. Genetic testing revealed a heterozygous truncating frameshift variant in the *ADNP* gene (c.104_105del; p.Ile35Argfs*4), predicted to undergo nonsense-mediated decay (Table 2, Figure 2).

### 3.3. Patient #3

A 5-year-old male, born late preterm, presented with global developmental delay and moderate intellectual disability. Motor milestones were delayed, and expressive language was limited to short phrases. Dysmorphic features included flat facial profile, synophrys, downslanting palpebral fissures, low-set ears, long philtrum, and widely spaced teeth. Additional findings included genu valgum, sacral dimple, and obesity (Figure 1j–n). Cardiac evaluation demonstrated septal hypertrophy. Behavioral features included hyperactivity, attention deficit, mouthing behavior, self-injury, and limited symbolic play. Ophthalmologic evaluation revealed severe hyperopia and strabismus. Genetic analysis identified a heterozygous nonsense variant in the *ADNP* gene (c.1216C>T; p.Gln406*), located in exon 6 and not predicted to undergo nonsense-mediated decay (Table 2, Figure 2).

### 3.4. Patient #4–5–6

This family included three individuals carrying the same heterozygous frameshift variant in the *ADNP* gene (c.2815_2816del; p.Ile939Serfs*4), located in the terminal exon and not predicted to undergo nonsense-mediated decay, demonstrating clear familial segregation (Figure 2).

The proband (P#4), a 2.5-year-old male, presented with a severe phenotype characterized by global developmental delay, hypotonia, severe epilepsy, marked microcephaly, and facial dysmorphism (Figure 1o,p). Brain MRI revealed periventricular leukomalacia, ventriculomegaly, corpus callosum thinning, and hippocampal atrophy. He had a history of severe perinatal hypoxia (HIE stage 3) requiring prolonged neonatal intensive care. Additional findings included congenital heart defects, genitourinary anomalies, and possible autistic features, although formal assessment was limited.

In contrast, P#5, a 17-year-old sister, exhibited a markedly milder phenotype, with no reported neurodevelopmental delay, major organ anomalies or major facial dysmorphism (Figure 1q,r). Her medical history included oligohydramnios and early tooth eruption. Although she was born after a difficult delivery, it proceeded without complications; APGAR scores were normal, and transfontanelle cranial ultrasonography revealed no anatomical abnormalities or bleeding inside the cranium. Furthermore, during early infancy, there were no central nervous system signs, neurodevelopmental delays, or any other findings attributable to the syndrome. In addition, a cranial MRI performed during adolescence due to an episode of facial paralysis was reported as normal.

P#6, a 47-year-old male and the father of P#4 and P#5, was almost asymptomatic, with no neurodevelopmental impairment or recognizable dysmorphic features (Figure 1s,t). Aside from obesity, his clinical evaluation was largely unremarkable. Interestingly, the older brother, who did not have the variant, also had a history of early tooth eruption.

Detailed characteristics of the cohort are depicted in Table 1 and Table 2.

## 4. Discussion

In this study, we present the first Turkish series of patients with *ADNP*-associated neurodevelopmental disorder, expanding both the genetic and phenotypic spectrum of Helsmoortel–Van der Aa syndrome. All identified variants were heterozygous and predicted to result in premature truncation of the ADNP protein, consistent with the established loss-of-function disease mechanism. Notably, none of the variants represented recurrent hotspot mutations, and three were novel, further supporting the allelic heterogeneity of *ADNP*-related disease. Our cohort also highlights substantial intrafamilial variability, ranging from severe neurodevelopmental impairment with epilepsy and structural brain abnormalities to nearly asymptomatic individuals, underscoring the broad expressivity associated with *ADNP* variants.

Although *ADNP*-associated disorder is most often caused by de novo truncating variants, our findings provide evidence for familial transmission with reduced penetrance and variable expressivity. A particularly important finding in our series is the presence of familial cases (P#4–5–6) carrying the same truncating variant. While P#4 exhibited a severe phenotype with epilepsy, structural brain abnormalities, and multisystem involvement, P#5 showed minimal or no neurodevelopmental impairment and P#6 was nearly asymptomatic. This striking intrafamilial variability reinforces the concept of variable expressivity and reduced penetrance in *ADNP*-related disorders. Also, the fact that the same disease-causing *ADNP* variant was identified in two siblings as well as in their clinically very mildly affected father indicates that the presence of a truncating *ADNP* variant alone may not be sufficient to determine clinical severity, and that modifier genes, epigenetic regulation, mosaicism, or environmental factors may play a role in phenotypic expression. This has important implications for variant interpretation and genetic counseling. Likewise, Van Dijck et al. reported probands diagnosed with *ADNP*-related HVDAS who inherited a pathogenic variant from an unaffected parent [5]. This study represents the third molecularly confirmed individual with this disorder, to the best of our knowledge. This finding underlines the importance of parental testing to evaluate their genetic status and inform recurrence risk assessment. Nevertheless, it should be acknowledged as a study limitation that P#5 and P#6 did not undergo standardized cognitive assessments, and the clinical history for P#6 was largely based on verbal history.

Another notable observation in our cohort is the prolonged retention of the umbilical cord, identified in two unrelated patients and complicated by infection. Delayed cord separation is classically associated with defects in neutrophil function or immune dysregulation, and delayed wound healing or frequent infections have been previously reported in the literature for HVDAS; however, the specific occurrence of delayed or fragmented umbilical cord separation accompanied by infection has not been documented [10,11]. This unique clinical presentation constitutes a novelty of our study, but to generalize, more robust data with larger patient groups are needed. Given the role of the ADNP protein in chromatin remodeling and broad transcriptional regulation, it is plausible that downstream effects on immune pathways could contribute to this phenotype. Nevertheless, this observation should be interpreted cautiously, and additional reports are required to determine whether this represents a recurrent or coincidental finding.

A growing body of evidence indicates that pathogenic variants in the *ADNP* gene are not randomly distributed but rather cluster in recurrent mutational hotspots. Frequently reported variants include p.Tyr719*, p.Arg730*, p.Asn832Lysfs*81, p.Val180Glyfs*17, and p.Val180Glyfs*81, which together account for a substantial proportion of published cases and have contributed to emerging genotype–phenotype correlations [11,12]. Notably, none of these recurrent hotspot variants were identified in our cohort, which instead comprised distinct truncating variants (Table 2). Nonsense-mediated mRNA decay (NMD) is a conserved mRNA surveillance pathway that degrades transcripts harboring premature termination codons (PTCs) to prevent the translation of potentially deleterious truncated proteins. NMD susceptibility is canonically predicted based on positional boundaries, wherein PTCs located more than 50–55 nucleotides upstream of the final exon-exon junction trigger degradation [13]. Except for the c.104_105del p.(Ile35Argfs*4) variant, all identified truncating variants fell outside these canonical boundaries (residing in the penultimate or terminal exons) and were predicted to escape NMD. However, ADNP transcript abundance was not experimentally evaluated, which constitutes a limitation of our predictive assessment.

Importantly, the molecular consequences of the truncating variants identified in our cohort warrant a more nuanced interpretation of the established loss-of-function model. Although haploinsufficiency has been considered a major mechanism underlying *ADNP*-related disorder, three of the four distinct variants identified in our cohort are predicted to escape nonsense-mediated mRNA decay (NMD), including the variants identified in the familial P#4–6 cluster. NMD escape does not necessarily imply preservation of normal ADNP function, as these variants may produce truncated proteins with altered stability, localization, protein–protein interactions, or transcriptional activity. Thus, the pathogenic effects of NMD-escaping variants may involve mechanisms beyond simple reduction in functional protein dosage, including altered or potentially dominant effects of the truncated protein. Notably, the same NMD-escaping c.2815_2816del (p.Ile939Serfs*4) variant was associated with markedly different clinical manifestations in our family, ranging from severe neurodevelopmental impairment to an apparently asymptomatic carrier, further indicating that NMD status alone is insufficient to predict phenotypic severity. Our findings therefore support a model in which loss of ADNP function remains an important component of disease pathogenesis, while the biological consequences of individual truncating variants and additional genetic, epigenetic, or environmental modifiers may contribute to the substantial clinical variability observed in *ADNP*-related disorder. Functional studies assessing transcript stability, residual protein production, subcellular localization, and transcriptional activity will be necessary to clarify the precise mechanisms associated with NMD-escaping variants.

The localization of pathogenic variants reported on *ADNP* has been categorized into two distinct groups based on previously defined DNA methylation episignatures: those associated with CpG hypomethylation (epi-ADNP-1) and those associated with CpG hypermethylation (epi-ADNP-2) [12]. All variants identified in this study belong to the CpG hypomethylating group and are located in regions proximal to either the N- or C-terminus. With the exception of the c.2491_2494del (p.Leu831Ilefs*82) variant (ClinVar ID: 139631; this variant was also detected in one person in gnomAD with 6.195 × 10^−7^ allele frequency), none of the other variants have been previously reported in databases and were absent from gnomAD and therefore considered novel (Table 2). According to consensus annotations in public protein databases, the ADNP sequence contains several well-defined functional domains: nine Cys2His2-type zinc-finger (C2H2-ZF) motifs (residues 74–97, 107–129, 161–188, 221–244, 446–469, 488–510, 512–535, 621–647, 660–686) mediating DNA binding and transcriptional regulation, the NAPVSIPQ octapeptide (NAP motif; residues 354–361) driving microtubule interaction and cellular neuroprotection, a bipartite nuclear localization signal (NLS; residues 714–733) directing the translocation of the protein into the nucleus, a homeodomain (residues 757–818) facilitating sequence-specific DNA binding, and a PxVxL motif (residues 820–824) mediating the interaction with heterochromatin protein 1 (HP1). Interestingly, none of the identified variants were located in functional domains of the ADNP protein. However, premature truncation results in the substantial downstream loss of critical functional domains. Specifically, the N-terminal p.(Ile35Argfs*4) variant leads to the complete loss of downstream functional architecture, including all nine C2H2-zinc finger motifs, the neuroprotective NAP motif (residues 354–361), the bipartite nuclear localization signal (NLS; residues 714–733), the homeobox domain (residues 757–818), and the PxVxL HP1-interacting motif (residues 820–824). In contrast, downstream truncating variants (e.g., p.Gln406*) retain the N-terminal zinc fingers and the NAP motif but lead to the loss of the C-terminal NLS, homeodomain, and PxVxL motif, potentially compromising nuclear import and chromatin-remodeling functions (Figure 2).

From a genotype–phenotype perspective, no clear correlation between the position of variants within the *ADNP* gene and clinical severity was observed in this cohort. Variants were distributed across different regions of the gene, and both early and late truncating variants were associated with a wide range of clinical outcomes, including severe neurodevelopmental impairment, obesity, epilepsy, and in some cases minimal or absent clinical manifestations. This observation aligns with previous reports suggesting that *ADNP*-related disease does not follow a simple positional effect model.

Helsmoortel–Van der Aa syndrome is a rare yet often severe condition, and its long-term prognosis remains insufficiently defined. Current management is largely supportive and directed toward symptom control, as no established disease-modifying therapy is available. Nevertheless, emerging evidence from preclinical studies and early clinical investigations suggests potential therapeutic avenues. In particular, Davunetide (NAPVSIPQ), a neuroprotective peptide derived from the *ADNP* gene, has demonstrated promising effects through its action on cytoskeletal stabilization [14]. In addition, low-dose ketamine has been associated with clinical improvement in an open-label study involving children with this disorder, possibly mediated by upregulation of ADNP expression; however, these findings require further validation in larger, controlled studies [15].

## 5. Conclusions

Overall, our findings support the concept that *ADNP*-associated neurodevelopmental disorder is a genetically heterogeneous condition with incomplete penetrance and highly variable expressivity, even among individuals carrying identical variants in the same family. Recognition of inherited *ADNP* variants in asymptomatic or mildly affected individuals underscores the need for cautious interpretation of pathogenic variants and highlights the importance of family-based genetic evaluation. Future studies integrating functional assays and broader genomic contexts will be essential to elucidate the mechanisms underlying phenotypic variability in *ADNP*-related disease.

## Figures and Tables

**Table 1:** The clinical characteristics of the cohort.

Patient ID	Age (years)/ Sex	Age at Diagnosis (years)	Prenatal History	Growth Parameters	Neurological Features	Dysmorphism	Cardiac Findings	Imaging	Genitourinary	Psychiatric/ Behavioral	Other
P1	13.5/F	11	N	W: 115 kg (SD: +5.61) L: 153 cm (SD: −0.71) HC: 59.5 cm (SD: +3.53)	Motor delay (walking 3.5 years, speech delay, no sentences, two-three words)	Low anterior hairline, depressed nasal bridge, short nose, wooly hair, hypertelorism, sparse eyebrows, bilateral epicanthus, mild eversion of lateral third of lower eyelids, narrow palpebral fissures, prominent-everted ear lobules, long philtrum, everted lower lip vermilion, downslanted upper lip vermilion, midface hypoplasia, downturned oral commissures, thin upper lip, short neck, left single palmar crease, brachydactyly of hands and feet, pes planus	Secundum atrial septal defect (closed with surgery)	Nonspecific diffusion restriction	N	Hand stereotypies, Self-harm, hand biting	Obesity, metabolic syndrome, hepatomegaly, hepatosteatosis, prolonged umbilical cord retention complicated with infection
P2	4/M	2	Increased risk in screening	W: 16 kg (SD: −0.4) L: 100 cm (SD: −0.99) HC: 48 cm (SD: −2.04)	Borderline motor delay (crawling on hands and knees 14–16 mo, walking 2.5 years), speech delay (no words at last visit, nonspecific babbling), special education	Mild scaphocephaly, prominent forehead, short nose with anteverted nares, sparse medial eyebrows, low-set posteriorly rotated ears with prominent earlobes, long smooth philtrum, thin upper lip, widely spaced teeth, full cheeks, partial 2–3 toe syndactyly, bifid teeth later fused, fetal finger pads,	Aortic valve abnormalities (bicuspid valve), Patent foramen ovale	Ventriculomegaly, delayed myelination, posterior white matter hyperintensity	Suspected, bilateral inguinal hernia, Orchiopexy for undescended testes, recurrent URTIs, scrotal hypoplasia, micropenis (buried?)		Early tooth eruption, nasolacrimal duct obstruction, prolonged umbilical cord retention with infection requiring hospitalization at one month due to infection (omphalitis), cardiac arrest, frequent infection, older brother receiving speech therapy at age 6 without diagnosis
P3	5/M	4.5	Late preterm (36 w)	W: 23 kg (SD: +1.91) L: 102 cm (SD: −1.01) HC: 50 cm (SD: −0.88)	GDD/ID, motor and speech delay (walking 20 mo), speech delay(15–20 words at last visit, 2–3-word sentences)	Strabismus, synophrisis, downslanting palpebral fissures, flat face, short nose, flat nasal bridge, low-set ears, long philtrum, widely spaced teeth, sacral dimple, genu valgum	Cardiac septal hypertrophy	N/A	Scrotal hypoplasia	Limited symbolic play, hyperactivity attention deficit disorder, happy demeanor; frequent mouthing behavior, self-harm, not compatible with the autism spectrum	Obesity, severe hyperopia (+7D), intermittent vomiting
P4	2.5/M	1.5	N	W: 15 kg (SD: +1.02) L: 90 cm (SD: −0.02) HC: 43 cm (SD: −4.26)	Severe epilepsy, hypotonia, GDD/ID, motor and speech delay	Strabismus, upslanting palpebral fissures, low-set ears, mildly high-arched palate	Patent foramen ovale, septal hypertrophy, thin ductus arteriosus	Periventricular leukomalacia, ventriculomegaly, corpus callosum thinning, hippocampal atrophy	Bilateral hydronephrosis (grade I–II), epispadias, scrotal hyperpigmentation	Possible autistic features (limited object play, mild sensory/behavioral issues)	History of severe perinatal hypoxia/asphyxia (hypoxic–ischemic encephalopathy stage 3, cardiac arrest/CPR, 28-day NICU stay)
P5	17/F	17	Oligohydramnios, abnormal head shape (cephalohematoma) and fetal presentation at birth	W: 60 kg (SD: +0.40) L: 160 cm (SD: −0.46) HC: 53 cm (SD: −2.25)	N	Minor dysmorphism	N	N/A	N	Anxiety disorder	Early tooth eruption (4 months), history of facial palsy (age 8–10), history of bilateral pes valgus, nasolacrimal duct surgery, two episodes of unexplained fainting
P6	47/M	47	N	W: 116 kg (SD: +3.12) L: 175 cm (SD: −0.19) HC: 56 cm (SD: −1.13)	N	Minor dysmorphism	N/A	N/A	N	N	Mild Obesity

N: Normal; N/A: Not available; SD: Standard deviation; F: Female; M: Male; W: Weight; L: Length; HC: Head circumference; GDD/ID: Global developmental delay and intellectual disability.

**Table 2:** The genetic characteristics of the cohort.

ID	Variant (NM_001282531)/ACMG Classification	Exon	Functional Consequence	Predicted Episignature Group
P1	c.2491_2494del p.(Leu831Ilefs*82)/Pathogenic(PVS1, PS4, PM2)	Frameshift/Exon 6	Not predicted to undergo NMD	CpG hypomethylation
P2	c.104_105del p.(Ile35Argfs*4)/Likely Pathogenic (PVS1,PM2)	Frameshift/Exon 4	Predicted to undergo NMD	CpG hypomethylation
P3	c.1216C>T p.(Gln406*)/Likely Pathogenic (PVS1,PM2)	Nonsense/Exon 6	Not predicted to undergo NMD	CpG hypomethylation
P4	c.2815_2816del p.(Ile939Serfs*4)/Likely Pathogenic (PVS1,PM2)	Frameshift/Exon 6	Not predicted to undergo NMD	CpG hypomethylation
P5	c.2815_2816del p.(Ile939Serfs*4)/Likely Pathogenic (PVS1,PM2)	Frameshift/Exon 6	Not predicted to undergo NMD	CpG hypomethylation
P6	c.2815_2816del p.(Ile939Serfs*4)/Likely Pathogenic (PVS1,PM2)	Frameshift/Exon 6	Not predicted to undergo NMD	CpG hypomethylation

Bold/italic variants are novel changes.

## Data Availability

The raw data supporting the conclusions of this article will be made available by the authors on request.

## Abbreviations

The following abbreviations are used in this manuscript:

ABI	Applied Biosystems
ACMG	American College of Medical Genetics and Genomics
ADNP	Activity-Dependent Neuroprotective Protein
C2H2-ZF	Cys2His2 Zinc Finger
CES	Clinical Exome Sequencing
CpG	Cytosine–Phosphate–Guanine Dinucleotide
CPR	Cardiopulmonary Resuscitation
DNA	Deoxyribonucleic Acid
GDD	Global Developmental Delay
gnomAD	Genome Aggregation Database
HGMD	Human Gene Mutation Database
HIE	Hypoxic–Ischemic Encephalopathy
HP1	Heterochromatin Protein 1
HVDAS	Helsmoortel–Van der Aa Syndrome
IBS	Illustrator for Biological Sequences
MRI	Magnetic Resonance Imaging
NAPVSIPQ	Davunetide
NGS	Next-Generation Sequencing
NICU	Neonatal Intensive Care Unit
NLS	Nuclear Localization Signal
NMD	Nonsense-Mediated mRNA Decay
